# Adenosine-A2 Receptor Signaling in Astrocytes Mitigates Cerebral Ischemic Injury via Suppressing Glutamate-Induced Excitotoxicity

**DOI:** 10.3390/cells15201915

**Published:** 2026-10-21

**Authors:** Cheng Yuan, Jixin Chen, Yazhi Chen, Liyuan Cui, Yingjiao Liu

**Affiliations:** 1 College of Pharmacy, Jiangxi University of Chinese Medicine, Nanchang 330004, China; yuancheng@jxutcm.edu.cn (C.Y.); chenjixin@jxutcm.edu.cn (J.C.); chenyazhi1@jxutcm.edu.cn (Y.C.); 2 State Key Laboratory of Bioactive Substances and Functions of Natural Medicines, Institute of Materia Medica & Neuroscience Center, Chinese Academy of Medical Sciences and Peking Union Medical College, Beijing 100050, China; 15850677252@163.com

**Keywords:** adenosine, stroke, astrocytes, excitotoxicity, A_2A_ receptor, A_2B_ receptor

## Abstract

**Highlights:**

**What are the main findings?**

- This study elucidates a novel astrocyte-centered mechanism whereby adenosine attenuates ischemic excitotoxicity by modulating the glutamate–glutamine cycle.
- Adenosine regulates glutamate transport and extracellular diffusion.
- The adenosine–A2AR system maintains glutamate homeostasis.

**What are the implications of the main findings?**

- A rat thromboembolic stroke model established via middle cerebral artery occlusion with autologous blood clots more accurately recapitulates the pathophysiology of human ischemic stroke and enhances clinical translatability.
- Optogenetic manipulation of astrocytes, with light-sensitive opsin expression driven by the GFAP promoter, validates their pivotal regulatory role in cerebral ischemic injury, delineates the functional contribution of astrocyte–neuron crosstalk in embolic stroke, and provides mechanistic evidence supporting astrocyte-targeted therapeutic strategies for ischemic stroke.

**Abstract:**

Excitotoxicity constitutes a core pathological mechanism in cerebral ischemia and other central nervous system disorders. Astrocytes serve as pivotal regulators of glutamate–glutamine cycle homeostasis and thereby play a critical role in mitigating excitotoxicity. This study established a rat thromboembolic stroke model via autologous thrombus induction and systematically investigated the mechanisms underlying adenosine-mediated neuroprotection through pharmacological modulation of the adenosine pathway using adenosine, dipyridamole, and selective adenosine receptor antagonists. Subsequently, light-sensitive opsin expression in astrocytes was driven by the GFAP promoter, enabling cell-type-specific optogenetic modulation of astrocyte activity. Our findings demonstrate that adenosine significantly attenuates cerebral injury in a rat model of thromboembolic stroke, with astrocytes identified as the primary cellular target. Adenosine elicits an anti-excitotoxic effect by coordinately upregulating GLT-1 protein expression and downregulating Cx43 protein expression in astrocytes, thereby regulating glutamate transport and diffusion. Moreover, our findings demonstrate that adenosine-mediated neuroprotection is significantly attenuated by SCH58261 but not by PSB1115. We conclude that the adenosine–A_2A_ receptor system plays a critical role in the regulation of glutamate homeostasis.

## 1. Introduction

Stroke is an acute cerebrovascular disease and has become the leading cause of disability worldwide. In China, stroke incidence is shifting toward younger populations, with over 66.6% of first-ever stroke cases occurring in adults aged ≤55 years [1]. Ischemic stroke (IS) is the most common type of stroke, significantly affecting human health due to its high incidence, disability rate, and mortality [2]. Glutamate serves as the primary excitatory neurotransmitter in the central nervous system (CNS), critically regulating neural transmission, activity-dependent synaptic plasticity, and cognitive functions. However, under pathological conditions such as cerebral ischemia, extracellular glutamate levels can rise to hundreds of micromolar, triggering excitotoxicity. Excessive glutamate overactivates N-methyl-D-aspartate receptors (NMDARs), thereby disrupting Ca^2+^ homeostasis and culminating in cytotoxic edema and neuronal death [3]. Glutamate transporters (GLT-1/EAAT2) are primarily expressed in astrocytes and mediate the overall clearance of extracellular glutamate at synapses, preventing excitotoxicity and facilitating the glutamate–glutamine (Glu–Gln) cycle [4]. Therefore, GLT-1 plays a crucial role in regulating glutamate homeostasis. Gap junction (GJ) proteins also affect the Glu–Gln cycle. Astrocytes establish extensive intercellular networks in the CNS via GJs and hemichannels, wherein connexin 43 (Cx43) serves as the predominant astrocytic connexin isoform [5]. Under normal physiological conditions, Cx43 hemichannels remain closed, allowing astrocytes to rapidly clear glutamate released into the synaptic cleft via efficient transporters, thereby maintaining glutamate homeostasis. Under pathological conditions such as cerebral ischemia, Cx43 expression is abnormally upregulated and its phosphorylation status altered, leading to persistent abnormal opening of hemichannels and excessive release of glutamate into the extracellular space, thereby disrupting the Glu–Gln cycle [6]. Therefore, enhancing glutamate uptake and conversion, as well as inhibiting glutamate diffusion, are potential strategies for preventing excitotoxicity.

Adenosine, an endogenous nucleoside, serves as a critical neuromodulator in the CNS and exhibits potent neuroprotective effects within the brain [7]. Under physiological conditions, adenosine modulates neuronal excitability and synaptic plasticity, thereby maintaining brain microenvironmental homeostasis and normal neuronal function. During ischemia–hypoxia, rapid ATP degradation occurs to restore energy homeostasis, leading to a significant elevation in adenosine levels. This increase promotes neuronal survival under conditions of reduced energy supply by dampening excitatory stimuli [8]. Enhancing endogenous adenosine levels has long been a pivotal therapeutic strategy for CNS disorders, exemplified by the use of adenosine kinase inhibitors [9] and dipyridamole [10]. However, studies have reported [11] that recurrent hypoxia attenuates adenosine production, leading to a progressive depletion of available adenosine. The depletion of endogenous adenosine heightens the susceptibility of brain neurons to ischemia and hypoxia. Therefore, it is imperative to administer exogenous adenosine to counteract endogenous adenosine depletion. Sustained activation of neuroprotective pathways via the administration of exogenous adenosine to maintain extracellular adenosine levels represents a promising therapeutic strategy [12,13]. However, the mechanisms underlying its efficacy against ischemia-induced excitotoxicity remain poorly elucidated. In fact, many functions of adenosine are mediated through its receptors, among which the A_1_ and A_2A_ (A_2A_R) receptors exhibit high affinity and are highly expressed in the CNS. Ruan et al. [14] suggested that activating adenosine A_1_R exerts neuroprotective effects in the OGD/R model. However, due to the numerous cardiovascular side effects associated with A_1_R activation, its application is also limited. Indeed, extracellular adenosine concentrations increase substantially following ischemic stroke, resulting in preferential activation of the A_2A_R and concomitant attenuation of A_1_R-mediated signaling [15]. It is worth noting that in the complex network of the neurovascular unit, the OGD/R model does not simulate the presence of adenosine, which affects adenosine receptors in a concentration-dependent manner. Moreover, A_2A_R plays different roles at various stages of ischemia, exerting both neuroprotective and neurotoxic effects on neurons [16]. However, its specific roles in astrocytes remain poorly characterized and warrant further mechanistic investigation. Moreover, adenosine A_2A_R forms heterodimers with A_2B_R, thereby impairing A_2A_ ligand binding and downstream signal transduction [17]. However, the functional relevance of this interaction in ischemic stroke remains unestablished, and adenosine exhibits low intrinsic affinity for A_2B_R. Currently, most studies primarily focus on neurons and target the corresponding receptors through blockers or agonists. This study primarily focuses on astrocytes as target cells, investigating how adenosine modulates the Glu–Gln cycle through its receptors to exert an antagonistic effect against excitotoxicity, thereby providing insights into the mechanism by which adenosine acts in excitotoxicity during ischemic stroke.

## 2. Materials and Methods

### 2.1. Experimental Reagents

Adenosine (A010255) and dipyridamole (A010123) were provided by Zhuhai Anze Biotechnology Co., Ltd., Zhuhai, China. SCH58261 (WDGQD) and PSB1115 (WGRTJ) were both provided by Sparkjade®, Jinan, China. EAAT2 (3k59182), Cx43 (8k9296), and β-actin (12w2944) were provided by Affinity Biosciences Group Ltd., Liyang, China. Stripping buffer (Solarbio^®^, Beijing, China, SW3022), GFAP (10024116, Proteintech, Wuhan, China), goat anti-rabbit IgG (Cat. No. AS014, ABclonal, Woburn, MA, USA), Alexa Fluor 488-conjugated goat anti-rabbit IgG (H+L) secondary antibody (Cat. No. AC231007003, Servicebio, Wuhan, China), Dnk pAb to Ms IgG Alexa Fluor^®^ 555 (Cat. No. 1005780-6, Abcam), HBAAV2/9-GFAP-hChR2 (H134R)-mCherry (91052424), and HBAAV2/9-GFAP-eNpHR3.0-E (109112501) were provided by Hanbio, Shanghai, China. TTC (2,3,5-triphenyltetrazolium chloride, Cat. No. 3550506004, Solarbio^®^), CheKine^TM^ Glutamate (Glu) Content Detection Kit (KTB1440), and CheKine^TM^ Glutamine (Gln) Content Detection Kit (KTB1441) were provided by Abbkine, Wuhan, China. Adenosine standard (110879-201703) was provided by the National Medical Products Administration, and BCA kit (Cat. No. PC0020, Solarbio^®^).

### 2.2. Experimental Animals

Male SPF-grade SD rats (weighing 220 ± 10 g) were purchased from Hunan Silaikejingda Experimental Animal Co., Ltd., Changsha, Hunan, China (License No. SYXK(Xiang)2021-0002). All experimental procedures were approved by the Experimental Animal Science Center of Jiangxi Chinese Medicine University (JZLLSC20250537). Animals were housed under pathogen-free conditions (temperature, 25 °C; relative humidity, 40–60%), were maintained on a 12/12 h light/dark cycle, and had free access to food and water. Rats were randomly divided into the following groups for the experiments discussed in the corresponding Results subsections:

• Section 3.1: Sham, Model, Adenosine (Ade);
• Section 3.2: Sham, Model, Dipyridamole (Dip), Ade, Dip+Ade;
• Section 3.3: Sham, Model, Ade, A_2A_R inhibitor (SCH58261), Ade + SCH58261, A_2B_R inhibitor (PSB1115), Ade + PSB1115;
• Section 3.4: Ade+optogenetic inhibition (Ade + Opto inh), Opto inh, Ade + optogenetic activation (Ade + Opto act), Opto act.

Each group consisted of 10 rats.

### 2.3. Establishment of a Thromboembolic Stroke Model

After a seven-day acclimation period, rats were anesthetized by inhalation of 3% isoflurane for induction, followed by continuous maintenance of anesthesia with 1.5% isoflurane. The Sham group underwent suturing after vascular dissection, while the Model group and drug treatment group received complete surgery to establish the thromboembolic stroke model (TE model). Rats were positioned supine on a surgical board, and a 2 cm longitudinal incision was made in the ventral cervical skin to separate the external carotid artery (ECA), internal carotid artery (ICA), and common carotid artery (CCA). An arterial clamp was used to occlude the ECA. The CCA was positioned within the groove of the vascular electrode clamp of a small-animal thrombosis generator (YLS-14B; Jinan Yiyan Science & Technology Development Co., Ltd., Jinan, China). The clamp was adjusted to an appropriate height and angle, followed by a 1 min pre-stimulation at a current intensity of 1.20 mA. Then, the thrombus was pushed into the ICA and the ICA was clamped, followed by continuous stimulation for 4 min. After the stimulation ended, the electrode clamp was removed, and a black thrombus approximately 1 cm long was observed within the CCA. The thrombus was then gently fragmented ten times using fine-tipped, non-traumatic forceps, resulting in its rapid embolization into the ICA. Then, the proximal end of the CCA was clamped for 10 min. After completion, all arterial clamps were removed, and the incision was closed. Postoperatively, the rats’ body temperature was maintained at 36.7–37 °C using a temperature-controlled heating pad. Cerebral perfusion was monitored using an EX13 5HU laser Doppler device (Moor Instruments, Axminster, UK) to determine the success of the occlusion. Rats showing persistent ischemia before embolization and with a baseline below 25% were included in the model [18]. This embolic stroke model closely recapitulates the pathophysiology of human thromboembolic ischemic stroke.

### 2.4. Drug Administration

Five days before surgery, rats were administered Dip solution by intramuscular injection twice daily at a dose of 0.25 mg/kg. Adenosine solution was administered immediately after surgery via tail vein injection at a dose of 20 nmol/kg. Prior to surgery, the inhibitor was administered. Rats were then positioned supine on a stereotaxic frame (RWD 68001, RWD Life Science Co., Ltd., Shenzhen, China), and a midline longitudinal incision was made through the scalp to expose the skull. The anterior fontanelle was used as the stereotaxic reference point. The lateral ventricle was selected as the injection site (AP: −0.60 mm, L: −1.5 mm, D: −4.0 mm). The inhibitor was injected using a microinjection pump (TFD03-01, Leifu Fluid (Baoding) Intelligent Equipment Manufacturing Co., Ltd., Baoding, China), with SCH58261 at a concentration of 5 µmol/L and PSB1115 at 1 µmol/L. The needle was left in place for 3 min before being slowly withdrawn. Modeling was initiated immediately following wound closure by suturing.

### 2.5. Injection of Optogenetic Viruses

The optogenetic virus was stereotactically infused into the lateral ventricle three weeks prior to surgery. The viral construct for the activation group was HBAAV2/9-GFAP-hChR2(H134R)-mCherry (titer ≥ 2.00 × 10^12^ VG/mL); that for the inhibition group was HBAAV2/9-GFAP-eNpHR3.0-E (titer ≥ 2.00 × 10^12^ VG/mL). The steps for stereotactic brain injection are described in Section 2.4. Virus (2.5 µL) was loaded into a glass micropipette and delivered unilaterally into the target brain region at a controlled rate of 50 nL/min. The pipette was retained in situ for 3 min post-injection, followed by slow retraction. The ceramic pin was inserted vertically into the craniotomy site and firmly anchored using dental cement. Animal modeling was initiated three weeks post-surgery, and the optical fiber was immediately implanted into the ceramic cannula. Optogenetic stimulation was delivered using the RWD IOS-465-589 optogenetic inhibition system: 473 nm light for the activation group and 550 nm light for the inhibition group, with each session lasting 15 min (frequency: 20 Hz; pulse width: 10 ms; power: 20 mW).

### 2.6. Measurement of Infarct Volume and Edema Rate

Infarct volume and cerebral edema were quantified using TTC staining, as previously reported [19]. At 24 h post-modeling, rats were euthanized, and brains were immediately removed and placed on ice. The brains were then sectioned into six 2.5 mm thick coronal slices and stained with a freshly prepared 1.5% (*w*/*v*) TTC solution for 15 min at 37 °C in the dark. Infarct volume and cerebral edema were quantified using Image-Pro Plus 6.0 software (Media Cybernetics, Silver Spring, MD, USA). The infarct volume (%) was calculated as

(1) $$\frac{\left[infarcted\;brain\;volume\right]}{\left[contralateral\;intact\;hemisphere\;volume\times 2\right]}\times 100\%$$

The edema rate (%) was calculated as

(2) $$\frac{\left[infrarced\;brain\;volume-contralateral\;intact\;hemisphere\;volume\times 2\right]}{\left[contralateral\;intact\;hemisphere\;volume\times 2\right]}\times 100\%$$

### 2.7. Nissl Staining

Nissl staining selectively stains Nissl bodies in viable neurons, thereby enabling clear visualization of neuronal architecture and cellular integrity. The rat brain sections were deparaffinized and rehydrated, followed by staining with acid fuchsin solution for 20 min. Next, the sections were rinsed with PBS, differentiated with graded ethanol solutions, dehydrated through a series of increasing ethanol concentrations to absolute ethanol, cleared with xylene, and mounted with a rubber-based mounting medium. Specimens were then examined by transmission electron microscopy using an H-7650 microscope (Hitachi, Tokyo, Japan).

### 2.8. Magnetic Resonance Imaging

Magnetic resonance imaging (MRI) was performed 24 h after establishing the rat TE model. Rats were anesthetized with 1.5% isoflurane, immobilized in a dedicated small-animal MRI cradle equipped with adjustable dental incisor clamps and ear bars, and imaged using a 3.0 T MRI scanner (Philips Medical Systems International B.V., Best, The Netherlands). Brain imaging was performed using a surface radiofrequency (RF) coil placed over the rat’s head, and diffusion-weighted imaging (DWI) sequences were acquired. Infarct volumes were quantified using Image-Pro Plus 6.0 software by manually delineating regions of interest (ROIs) over high-signal areas on DWI scans. The infarction rate (%) was calculated as

(3) $$\frac{\left[ischemic\;hemisphere\;area\right]}{\left[2\times non\text{-}ischemic\;hemisphere\;area\right]}\times 100\%$$

The brain edema rate (%) was calculated as

(4) $$\frac{\left[ischemic\;hemisphere\;area-non\text{-}ischemic\;hemisphere\;area\right]}{\left[2\times non\text{-}ischemic\;hemisphere\;area\right]}\times 100\%$$

### 2.9. Western Blotting

After cell lysis, proteins were extracted for Western blotting. Following weighing, tissue samples were homogenized in cold RIPA lysis buffer and incubated on ice for 30 min. The lysate was centrifuged at 12,000 rpm for 30 min at 4 °C, and the supernatant was collected for protein quantification using the BCA assay. The sample was mixed with one-quarter volume of 5× SDS-PAGE loading buffer, and the mixture was heated at 100 °C to ensure denaturation. Equal amounts of protein (40 µg per lane) were resolved by 10% SDS-PAGE and electrotransferred onto PVDF membranes. Then, the membranes were blocked with blocking solution at room temperature for 30 min and subsequently incubated overnight at 4 °C with the following primary antibodies: β-actin (1:10,000), EAAT2 (1:3000), and connexin 43 (1:3000). After being washed three times with 1× TBST, the membranes were incubated with the secondary antibody (1:8000) at room temperature for 2 h. The protein expression levels were detected using an ECL ultra-sensitive chemiluminescence substrate and visualized with the Bio-Rad Gel Doc XR+ gel imaging system (Hercules, CA, USA). Densitometric analysis was performed using ImageJ 1.54g software (National Institutes of Health, Bethesda, MD, USA). Following Cx43 signal detection, the membrane was stripped of bound antibodies using stripping buffer. Following blocking, the membrane was incubated sequentially with an anti-β-actin primary antibody (1:10,000) and a secondary antibody, followed by visualization.

### 2.10. HPLC

HPLC analysis was conducted on a YMC-Pack ODS-AQ reversed-phase column (4.6 mm × 250 mm, 5 µm particle size) using an isocratic mobile phase of water–acetonitrile (91:9, *v*/*v*) at a flow rate of 1.0 mL/min. Detection was performed at 212 nm with the column temperature maintained at 30 °C. The adenosine reference standard was accurately weighed, dissolved in methanol, and diluted to volume with methanol to prepare a 409.2 µg/mL reference solution. Rat cortical tissue was isolated on ice and immediately snap-frozen in liquid nitrogen. A 0.4 mol/L perchloric acid solution containing 0.02% (*w*/*v*) ascorbic acid and 0.2 mmol/L EDTA-2Na was added to the tissue at a ratio of 10 mL per gram. The tissue was then homogenized thoroughly on ice and centrifuged at 15,000 rpm for 20 min at 4 °C, and the supernatant was collected. The sample was neutralized with 4 mol/L of KOH solution to pH 7.0–7.4 and centrifuged at 15,000 rpm for 20 min at 4 °C, and the supernatant was collected for subsequent analysis. Aliquots of 20 µL of the reference solution and sample supernatant were separately pipetted and then injected into a Waters e2695 high-performance liquid chromatograph for analysis.

### 2.11. Immunofluorescence

The animal was perfused transcardially under deep anesthesia, and then the brain was rapidly dissected and sectioned coronally at a thickness of 30 µm using a cryostat. The sections were heated by immersing them in pre-warmed citrate-based antigen retrieval buffer (pH 6.0) and then placing them in a microwave oven at 95–98 °C for 10 min, followed by passive cooling to room temperature. A 0.5% Triton X-100 solution was added to the sections, and they were incubated with gentle agitation for 15 min. Subsequently, nonspecific binding sites were blocked with 5% bovine serum albumin (BSA) in blocking buffer, followed by overnight incubation with the appropriate primary antibody at 4 °C. Following three washes with PBS, the sections were incubated with the appropriate secondary antibody for 2 h at room temperature. Nuclei were counterstained with DAPI, and slides were mounted with antifade mounting medium. Fluorescence images were acquired using a laser scanning confocal microscope (Carl Zeiss Microscopy GmbH, Jena, Germany, LSM 980).

### 2.12. Proteomics

After tissue lysis, equal amounts of total protein were extracted, precipitated by adding pre-chilled acetone, and incubated at −20 °C for 2 h. The mixture was then centrifuged to pellet the protein, and the supernatant was carefully discarded. The resulting pellet was washed 2–3 times with pre-chilled acetone. Following air-drying of the protein pellet at room temperature, TEAB buffer was added to a final concentration of 200 mM, and the sample was resuspended thoroughly by probe sonication. Trypsin digestion was then carried out overnight at 37 °C. Following enzymatic digestion, DTT was added to obtain a final concentration of 5 mM, and the mixture was incubated at 56 °C for 30 min to reduce disulfide bonds. Subsequently, iodoacetamide (IAA) was added to obtain a final concentration of 11 mM, and alkylation was performed in the dark at room temperature for 15 min. The resulting peptides were dissolved in solvent A (0.1% aqueous formic acid) and separated using a Vanquish Neo ultra-high-performance liquid chromatography (UHPLC) system. Mobile phase A consisted of 0.1% (*v*/*v*) formic acid in water; mobile phase B consisted of 0.1% (*v*/*v*) formic acid in 80% (*v*/*v*) acetonitrile/water. Chromatographic separation was performed at a flow rate of 600 nL/min using a predefined gradient program. Peptide fragments were ionized via capillary electrospray ionization and analyzed on a timsTOF HT mass spectrometer, with the source voltage set to 1.6 kV. Data acquisition was performed in dia-PASEF mode, with a full-scan MS1 range of 300–1500 *m*/*z*. Following each MS1 scan, 12 parallel accumulation–serial fragmentation MS2 events were triggered across the *m*/*z* range 395–1080, using a 15 *m*/*z* isolation window.

### 2.13. Dye Migration Experiment

Gap junction functionality is assessed based on the extent of dye diffusion within the cortical tissue. We analyzed the diffusion of lucifer yellow (LY), a permeability tracer for gap junction intercellular communication (GJIC), following its stereotaxic microinjection into the cortex of a rat TE model. Stereotaxic microinjection was performed with the following coordinates relative to bregma: anteroposterior, −0.6 mm; lateral, −1.5 mm; and ventral, −2.5 mm. A total of 2 µL of a solution containing lucifer yellow (5 µg/µL) was delivered into the right hemisphere at a constant rate of 0.25 µL/min. Twenty-four hours post-injection, animals were transcardially perfused with saline, and coronal brain sections (60 µm thick) containing the needle tract were collected. Intercellular fluorescent molecular diffusion was visualized using laser scanning confocal microscopy (LSCM; Leica, Wetzlar, Germany) and quantified in terms of diffusion distance using MetaMorph software (version 7.10.5, Molecular Devices, San Jose, CA, USA). Six coronal brain sections per rat were selected for analysis to ensure anatomical consistency.

### 2.14. ELISA

A fresh tissue sample weighing 0.1 g was maintained at 2–8 °C; 1 mL of physiological saline was then added, and the mixture was homogenized rapidly on ice. Following homogenization, tissue samples were centrifuged at 4 °C for 5 min at 8000× *g*, and the supernatant was carefully collected. Glu and Gln concentrations were quantified using a commercially available ELISA kit in accordance with the manufacturer’s protocol.

### 2.15. Statistical Analysis

All statistical analyses were performed using GraphPad Prism 7.0 software (La Jolla, CA, USA). All data are expressed as the mean ± standard deviation (mean ± SD), and *p* < 0.05 was considered statistically significant. Intergroup comparisons were performed using one-way analysis of variance followed by Tukey’s post hoc test.

## 3. Results

### 3.1. Adenosine Exerts a Significant Protective Effect Against Excitotoxicity Induced by Cerebral Ischemia

We established a TE model in rats via electrical stimulation. Animals in the Ade group received an intravenous injection of adenosine through the tail vein immediately after model induction and were euthanized 24 h post-induction for brain tissue collection (Figure 1A). Quantitative proteomic analysis was conducted on brain tissue samples from the Sham, Model, and Ade groups of rats, revealing statistically significant differences in global protein expression profiles across the three groups. Comparative quantitative proteomic analysis between the Model and Ade groups identified differentially expressed proteins, including Gja1 (Cx43), Slc1a2 (GLT-1), and GFAP (Figure 1B). These findings indicate that adenosine administration modulates the cerebral proteome in a rat model of cerebral ischemia. Differentially expressed proteins were significantly enriched in biological pathways governing glutamate transport and gap junction-mediated intercellular communication—functions canonically ascribed to astrocytes. Based on the above findings, TTC staining was performed to quantify cerebral infarct volume in the brain tissues of the three experimental rat groups (Figure 1C). The Model group exhibited markedly elevated cerebral infarct volume (30.2 ± 2.1%) and brain edema (15.4 ± 1.3%), both of which were significantly attenuated in the Ade group. Western blot analysis revealed that, relative to the Sham group, GLT-1 protein expression was significantly downregulated, and Cx43 protein expression was significantly upregulated in the Model group; these alterations were markedly attenuated by adenosine treatment (Figure 1D, Supplementary Information). Immunofluorescence colocalization analysis further confirmed that, relative to the Sham group, Cx43 protein expression was significantly upregulated on astrocytic membranes in the cortical region of the Model group, whereas GLT-1 expression was significantly downregulated; these changes were reversed in the Ade group (Figure 1E,F). Collectively, our findings demonstrate that adenosine exerts an anti-excitotoxic effect by increasing GLT-1 expression while simultaneously suppressing Cx43 protein expression.

### 3.2. Exogenous Adenosine Exerts a Predominant Neuroprotective Effect Against Excitotoxicity Relative to Endogenous Adenosine

Given the well-established neuroprotective role of adenosine in brain injury, we systematically compared the functional contributions of endogenous versus exogenous adenosine. To elevate endogenous adenosine levels in rat brain tissue, we administered dipyridamole (Dip); we then compared the extent of brain injury in the TE model across three experimental conditions: Dip-induced enhancement of endogenous adenosine, exogenous adenosine administration (Ade), and their combination (Dip+Ade) (Figure 2A). TTC staining (Figure 2B) revealed a significant reduction in both cerebral infarct volume and brain edema in the Dip group relative to the Model group. Compared with the Dip group, both the Ade and Dip+Ade groups exhibited further reductions in cerebral infarct volume and brain edema, with no significant difference between the two groups. As shown in Figure 2C, neurons in the right cerebral cortex of the Sham group exhibited homogeneous distribution, orderly laminar alignment, intact somatic morphology, plump cell bodies, and abundant, densely stained Nissl substance. In contrast, neurons in the Model group showed significant damage, with disordered cell arrangement, shrunken cell bodies, and extensive loss of Nissl bodies, resulting in a marked decrease in the density of surviving neurons. Neuronal pathology in the Dip group was attenuated relative to that in the Model group, whereas the Ade and Dip+Ade groups exhibited the most pronounced morphological preservation, with a progressive reduction in the extent of neuronal injury. These findings preliminarily indicate that exogenous adenosine is the primary mediator of neuroprotection. Western blot analysis revealed that both the Dip and Ade groups showed increased GLT-1 protein expression and decreased Cx43 protein expression, thereby enhancing glutamate uptake and suppressing glutamate release as well as the propagation of excitotoxicity. However, the Dip group exhibited a significantly weaker therapeutic effect compared with the Ade group, and no statistically significant difference was observed between the Ade and Dip+Ade groups (Figure 2D–F, Supplementary Information). Dye migration assays (Figure 2G–I) further demonstrated that the Ade group exhibited a significantly shorter dye diffusion distance than the Dip group, consistent with its inhibitory effect on Cx43 protein expression.

Additionally, adenosine levels in the rat brain tissue were quantified following cerebral ischemia (Figure 3A,B). Adenosine levels were markedly elevated in the Model group. In contrast, treatment with Dip, Ade, or Dip+Ade sustained the activation of adenosine receptors, thereby accelerating adenosine metabolism and leading to a significant reduction in adenosine levels. Subsequently, immunofluorescence colocalization analysis of A_2A_R with neurons (MAP-2) and astrocytes (GFAP) was performed. Analysis revealed significantly elevated neuronal A_2A_R protein expression following cerebral ischemia in rats, which was attenuated by Dip administration. Intervention with Ade or Dip+Ade resulted in marked upregulation of A_2A_R protein expression, accompanied by pronounced activation of astrocytes (Figure 3C). Dip inhibits nucleoside transporters (ENTs) to reduce adenosine reuptake [20,21], thereby elevating endogenous adenosine levels and downregulating the expression of A_2A_R and GFAP. Administration of exogenous adenosine in the Ade group upregulated A_2A_R expression and induced astrocyte activation, thereby exerting neuroprotective effects [11]. This implies that exogenous adenosine acts specifically via A_2A_R on astrocytes, a mechanism that is absent in endogenous adenosine signaling.

### 3.3. Adenosine Alleviates Cerebral Ischemia-Induced Excitotoxicity Through Activation of the A_2A_R Signaling Pathway

Previous studies have shown that adenosine can effectively counteract excitotoxicity, although the underlying mechanism remains unclear. Notably, research has already confirmed that adenosine receptors exert neuroprotective effects in brain injury [22,23]. Our prior study demonstrated that adenosine treatment significantly upregulated A_2A_R expression, leading us to hypothesize that the A_2A_R serves as the primary molecular target mediating adenosine’s neuroprotective effects. We administered the A_2A_R antagonists SCH58261 and PSB1115 via stereotaxic intracerebral injection, followed immediately by TE model induction (Figure 4A). The MRI findings (Figure 4B) revealed a significant increase in cerebral infarct volume in the Model group relative to the Sham group, which was markedly attenuated by adenosine treatment. Compared with the Model group, the Ade+PSB1115 treatment induced a significantly greater reduction in cerebral infarct volume than did the Ade+SCH58261 treatment. Nissl staining revealed that, relative to the Ade group, the Ade+SCH58261 group displayed intensified neuronal staining, pronounced cytoplasmic vacuolization, and a significantly elevated neuronal death rate; in contrast, the Ade+PSB1115 group exhibited only a marginal increase in neuronal death (Figure 4C).

Western blot analysis demonstrated that Ade administration upregulated GLT-1 and downregulated Cx43 protein expression, an effect that was not significantly altered by PSB1115 injection (Figure 5D–F, Supplementary Information). In contrast, SCH58261 treatment significantly attenuated the therapeutic effects of Ade (Figure 5A–C, Supplementary Information). The immunofluorescence results were consistent with the above findings. Colocalization analysis showed that, compared to the SCH58261 and Ade+SCH58261 groups, the Ade+PSB1115 group exhibited a significantly enhanced colocalization signal between GLT-1 and GFAP (Figure 5G), while the colocalization signal between Cx43 and GFAP was markedly reduced (Figure 5H). We further quantified Glu and Gln concentrations in the cerebral cortex of all seven experimental groups using ELISA (Figure 5I,J). The results revealed that cerebral ischemia induced a marked elevation in Glu and a significant reduction in Gln levels in the cerebral cortex of rats in the Model group. Adenosine treatment effectively reversed both alterations; however, co-administration of SCH58261 attenuated this protective effect, whereas PSB1115 exerted no statistically significant modulation. These findings indicate that pharmacological inhibition of the adenosine A2AR attenuates adenosine-mediated neuroprotection, whereas selective blockade of the A2BR fails to yield a statistically significant effect.

### 3.4. Optogenetic Manipulation Was Employed to Demonstrate That Adenosine Mediates Anti-Excitotoxic Effects Through Astrocytic Signaling

To further confirm the role of astrocytes in this process, optogenetic modulation with cell-type specificity was employed to selectively activate or inhibit astrocytes, thereby confirming their essential role in adenosine-mediated neuroprotection. The experimental workflow is schematically illustrated in Figure 6A. Western blot analysis revealed that selective optogenetic inhibition of astrocytes led to a significant decrease in GLT-1 protein expression and a significant increase in Cx43 protein expression in the rat cerebral cortex; conversely, optogenetic activation of astrocytes reversed both changes (Figure 6B, Supplementary Information). Quantitative immunofluorescence analysis further demonstrated that astrocytic silencing impaired glutamate transport. Astrocyte activation significantly upregulated glutamate transporter expression and reduced gap junction function; notably, co-administration of adenosine with optogenetic stimulation produced significantly greater effects than optogenetic stimulation alone (Figure 6C). This demonstrates that astrocytes are critically involved in adenosine-mediated regulation of glutamate transport.

## 4. Discussion

In ischemic stroke, acute occlusion of cerebral arteries precipitates a rapid reduction in cerebral perfusion, initiating a cascade of secondary brain injury, including excitotoxicity, oxidative stress, and disruption of ionic homeostasis [24]. Of these mechanisms, excitotoxicity represents the earliest and most pivotal pathological event in the ischemic cascade. The core pathophysiological mechanism involves excessive release of excitatory neurotransmitters—particularly glutamate—which causes sustained activation of postsynaptic excitatory receptors, thereby triggering intracellular calcium overload, oxidative stress, mitochondrial dysfunction, and neuroinflammatory responses. These interrelated processes collectively drive neuronal apoptosis and necrosis, culminating in persistent neurological deficits [25].

Adenosine is a naturally occurring nucleoside formed by the enzymatic degradation of ATP and regulates various physiological processes through activation of four G protein-coupled receptor subtypes—A_1_, A_2A_, A_2B_, and A_3_ [26]. Adenosine is classified as either endogenous or exogenous. Endogenous adenosine is synthesized and released by brain tissue under both physiological and pathological conditions, where it functions as an intrinsic neuroprotective mediator [27]. During cerebral ischemia, the imbalance in energy metabolism triggers a compensatory and rapid elevation of endogenous adenosine levels, which exerts neuroprotective effects via adenosine receptor signaling pathways. A substantial body of evidence indicates that adenosine exerts neuroprotective effects primarily through activation of the A_1_R [14,28], attenuation of Glu-mediated excitotoxicity [29], and induction of antioxidant responses coupled with inhibition of apoptotic pathways [30]. Administration of exogenous adenosine has been shown to augment and sustain neuroprotective effects, attenuate neuronal cell death, and facilitate functional neurological recovery [12]. However, this study demonstrated robust A_2A_R activation in both neurons and astrocytes within the thromboembolic ischemic stroke model in rats and further revealed differential modulation of A_2A_R by Dip and Ade. Exogenous adenosine preferentially activates A_2A_R on astrocytes and reverses the ATP–Ade cycle, thereby enhancing ATP synthesis to support the astrocyte-specific conversion of glutamate to glutamine. This mechanism likely underlies the observed reduction in tissue adenosine levels following exogenous adenosine administration.

Evidence indicates that A_2A_R activation modulates both the expression and functional activity of the glutamate transporter GLT-1 in traumatic brain injury (TBI) [31]. GLT-1 dysfunction accelerates excitotoxicity and neuronal damage. Neuronal A_2A_R upregulation has been documented in brain lesions from patients with Rasmussen encephalitis (RE), coinciding with GLT-1 downregulation [32]; pharmacological A_2A_R blockade attenuates excitatory neurotransmitter release and restores both GLT-1 expression and transport function [32,33]. However, co-administration of adenosine with SCH58261 markedly attenuated adenosine-mediated neuroprotection and concomitantly reduced GLT-1 protein expression in astrocytes; in contrast, no such effect was observed following administration of PSB1115. This observation appears to contrast with prior findings, a difference potentially attributable to model-dependent variations in adenosine concentration. Jones et al. [34] reported that intraperitoneal administration of adenosine, followed by a higher dose of an A_2A_R agonist (0.1 or 0.01 mg/kg), attenuated kainate-induced excitotoxicity in the hippocampus; in contrast, this neuroprotection was not observed at a lower dose (2 µg/kg). High-dose adenosine administration robustly activates adenosine A_2A_R [15], a mechanism that is proposed to mediate its neuroprotective effect against excitotoxicity. Prior studies have identified A_2A_R activation as a contributor to excitotoxic neuronal injury, leading to its characterization as a neurotoxic modulator [35]. However, Alessia et al. [36] demonstrated that the neuroprotective efficacy of A2AR antagonists is temporally confined to the first 24 h after cerebral ischemia and is not maintained beyond this acute phase. Moreover, A_2A_R activation has been shown to exert neuroprotective effects in certain contexts [37]. For example, A_2A_R activation has been shown to upregulate neurotrophic factor expression [12,38,39]; preserve blood–brain barrier integrity and inhibit cerebral metastasis of solid tumors [40]; protect neurons from excitotoxicity via astrocyte-specific A_2A_R signaling [41]; attenuate acute neurotoxic and pro-inflammatory responses following intracerebral hemorrhage [42]; slow disease progression in Huntington’s disease [43]; and confer neuroprotection in spinal cord injury through inhibition of JNK phosphorylation [44]. Excitotoxicity in IS is mechanistically linked to A2AR activation. Notably, A_1_R and A_2A_R engage in functional crosstalk through heterodimerization, and A_1_R activation has been shown to attenuate A_2A_R-mediated signaling responses. Whether adenosine levels modulate the A_1_R–A_2A_R interaction in ischemic stroke will be addressed in our upcoming studies.

Indeed, the dual functional roles of A_2A_R are context-dependent, critically governed by the pathological milieu, local concentrations of adenosine and glutamate, cellular identity, and the temporal profile of receptor activation, thereby positioning A_2A_R as a pivotal molecular switch in the regulation of excitotoxicity, cerebral ischemia, and neuroinflammation [45,46,47]. For instance, tumor cell-expressed A2AR drives tumor progression via adenosine-dependent signaling [48]; neuronal A_2A_R upregulation accelerates Alzheimer’s disease pathogenesis and exacerbates cognitive deficits [49], whereas neuron-specific A2AR knockdown attenuates fear memory impairments following traumatic brain injury [50]; activation of A2AR on M1 macrophages attenuates psoriatic inflammation [51]. A_2A_R highly expressed on neurons predominantly mediates neurotoxicity, whereas astrocytic A_2A_R exhibits context-dependent functional duality, exerting either neuroprotective or neurotoxic effects contingent upon activation intensity [52,53]. The A_2A_R agonist CGS21680 exerts neuroprotective effects upon transient, low-dose stimulation; however, prolonged or supraphysiological activation redirects its functional profile toward neurotoxicity [54,55,56]. This study further corroborates this finding. A breakthrough of this study is the identification of the protective role of astrocytic A_2A_R in cerebral ischemia. Our findings demonstrate that adenosine administration significantly upregulates GLT-1 protein expression and concurrently downregulates total Cx43 protein expression in the rat cerebral cortex; these effects are attenuated by pharmacological inhibition of A_2A_R. Adenosine likely attenuates excitotoxicity via astrocytic A_2A_R-dependent modulation of glutamate transport, metabolic conversion, and intercellular diffusion.

Under pathological conditions such as ischemia or neuroinflammation, transient closure or downregulation of Cx43-mediated gap junctional communication restricts the intercellular propagation of deleterious signals—including excitotoxicity and pro-inflammatory mediators—thereby conferring neuroprotection [57]. Studies report upregulation of Cx43 in the spinal cord of rodent models of neuropathic pain and spinal cord injury, indicating enhanced GJ-mediated intercellular communication between Cx43-expressing astrocytes and microglia. This facilitates the exchange of pro-inflammatory signaling molecules and thereby contributes to the chronicization of neuroinflammation. Pharmacological inhibition of the Sigma-1 R alleviates neuropathic pain through downregulation of Cx43 expression, leading to improvement of the neuroinflammatory microenvironment [58,59]. Compounds targeting Cx43 have shown promising preclinical efficacy. Gap19 exerts neuroprotective effects in the tMCAO model by activating the JAK2/STAT3 signaling pathway and confers anti-apoptotic protection through selective inhibition of astrocytic Cx43 hemichannels [6]. In the OGD/R in vitro model, carbenoxolone (CBX)-mediated inhibition of Cx43 channel opening has been shown to attenuate cerebral ischemic injury [60]. Phosphorylation serves as a pivotal regulatory mechanism for Cx43, directly governing the assembly and permeability of gap junction channels via modulation of its phosphorylation status [61]. Quantitative analysis of total Cx43 protein (NP-Cx43 + p-Cx43) revealed a significant upregulation of Cx43 expression in the right cerebral cortex of model rats, which was effectively attenuated by adenosine intervention. Adenosine effectively mitigates excitotoxicity via a multimodal mechanism involving the enhancement of glutamate uptake and metabolic clearance, alongside the suppression of its pathological diffusion. Moreover, the function of Cx43 also depends on its location. Upregulation of Cx43 at the astrocytic plasma membrane exacerbates neuronal damage in ischemic brain injury, whereas mitochondrial Cx43 (mtCx43) exerts neuroprotective effects during cerebral ischemia–reperfusion injury. mtCx43 mediates mitochondrial protection against cerebral ischemic injury via activation of the mitoKATP channel [62], providing novel insights into potential protective mechanisms against cerebral ischemia. Previous studies have demonstrated [63] that salvianolic acids (SAs) attenuate brain injury in both OGD in vitro models and MCAO rat models, primarily through PI3K/AKT-mediated upregulation of mtCx43.

To achieve more precise and efficient modulation of astrocytes and to further validate and identify superior therapeutic strategies, we employed optogenetics. This technique enables the selective regulation of astrocytic or neuronal activity, thereby exerting therapeutic effects against conditions such as epilepsy and stroke [64,65,66]. Studies have demonstrated [67] that the astrocyte-mediated antiepileptic effect of this intervention is underpinned by Na^+^−K^+^−ATPase activation, which stabilizes astrocytic intracellular Na^+^ gradients and enhances clearance of extracellular K^+^, thereby suppressing pathological neuronal hyperexcitability and seizure generation. Na^+^−K^+^−ATPase plays a pivotal role in the Glu–Gln cycle: by maintaining the transmembrane Na^+^/K^+^ electrochemical gradient, it provides the essential driving force for astrocyte-specific, Na^+^-coupled glutamate uptake, enabling subsequent intracellular conversion of glutamate to glutamine and thereby preventing excitotoxic neuronal injury. Optogenetics is an emerging, cutting-edge biotechnology that enables precise, cell-type-specific control of neuronal or glial activity [68,69]. Optogenetic targeting of astrocytes was achieved by packaging ChR2 and eNpHR3.0 into AAV vectors under the control of the GFAP promoter, thereby enabling cell-type-specific photoactivation or photoinhibition upon delivery of blue (473 nm) or yellow (593 nm) light, respectively. This study demonstrates, via astrocyte-specific optogenetic manipulation, that adenosine preserves Glu–Gln cycle homeostasis by activating astrocytic adenosine receptors, thereby modulating glutamate uptake, intracellular conversion to glutamine, and spatial redistribution of glutamate. Optogenetic technology holds significant translational promise for clinical diagnosis and therapy. When integrated with pharmacological interventions, it enables multimodal modulation of pathological neural circuits, thereby improving therapeutic efficacy and optimizing clinical outcomes.

## 5. Conclusions

Adenosine markedly attenuates neuropathological damage induced by cerebral ischemia. Its neuroprotective effects are primarily mediated by astrocyte-dependent regulation of extracellular glutamate homeostasis. The underlying mechanisms involve astrocyte-mediated regulation of glutamate uptake, enzymatic conversion to glutamine, and gap junction-dependent intercellular communication. Our experimental data demonstrate that adenosine confers neuroprotection primarily through activation of the astrocytic A2AR, thereby mitigating excitotoxic neuronal injury.

## Figures and Tables

**Figure 1:**
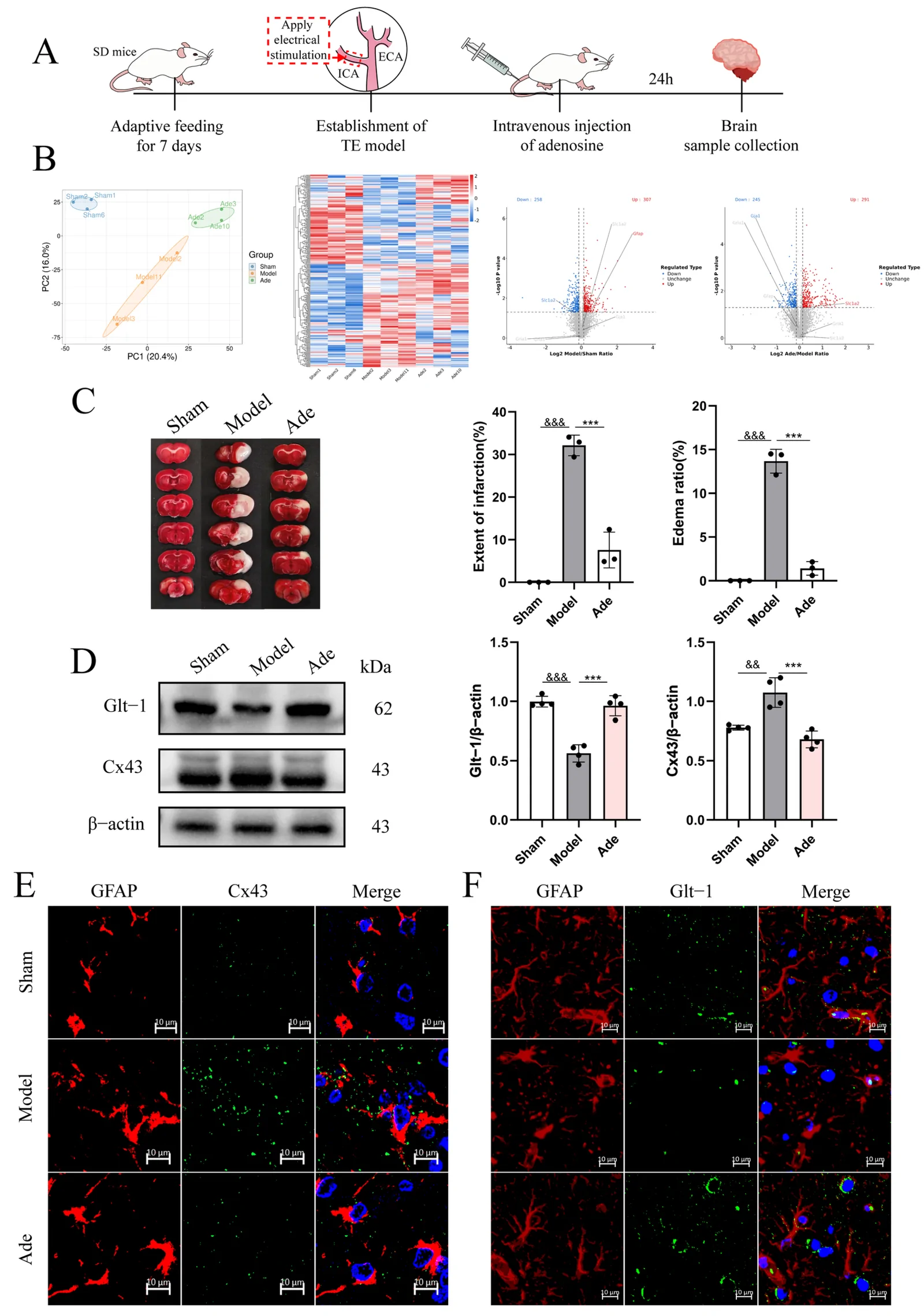
Adenosine significantly attenuates ischemia-induced excitotoxicity in the rat brain. (**A**) Schematic illustration of the experimental design and procedural workflow. (**B**) Principal component analysis (PCA) plot, hierarchical clustering heatmap, and volcano plot derived from quantitative proteomic profiling. (**C**) TTC staining results of rat brains in the Sham, Model, and Ade groups, along with quantitative analysis of infarct volume and edema rate; *n* = 3 independent samples. (**D**) Western blot analysis and quantification of GLT-1, Cx43, and β-actin protein levels in the right cerebral cortex of rats from the Sham, Model, and Ade groups. Data are representative of four independent experiments (*n* = 4). (**E**,**F**) Representative immunofluorescence images of the right cerebral cortex from the Sham, Model, and Ade groups. Nuclei were stained with DAPI (blue), astrocytes with GFAP (red), and Cx43 (**E**) or GLT-1 (**F**) is shown in green. Scale bar: 10 µm. Data are expressed as the mean ± SD. Statistical significance: vs. Sham, ^&&^ *p* < 0.01, ^&&&^ *p* < 0.001; vs. Model, *** *p* < 0.001. One-way ANOVA was performed with Tukey’s post hoc test.

**Figure 2:**
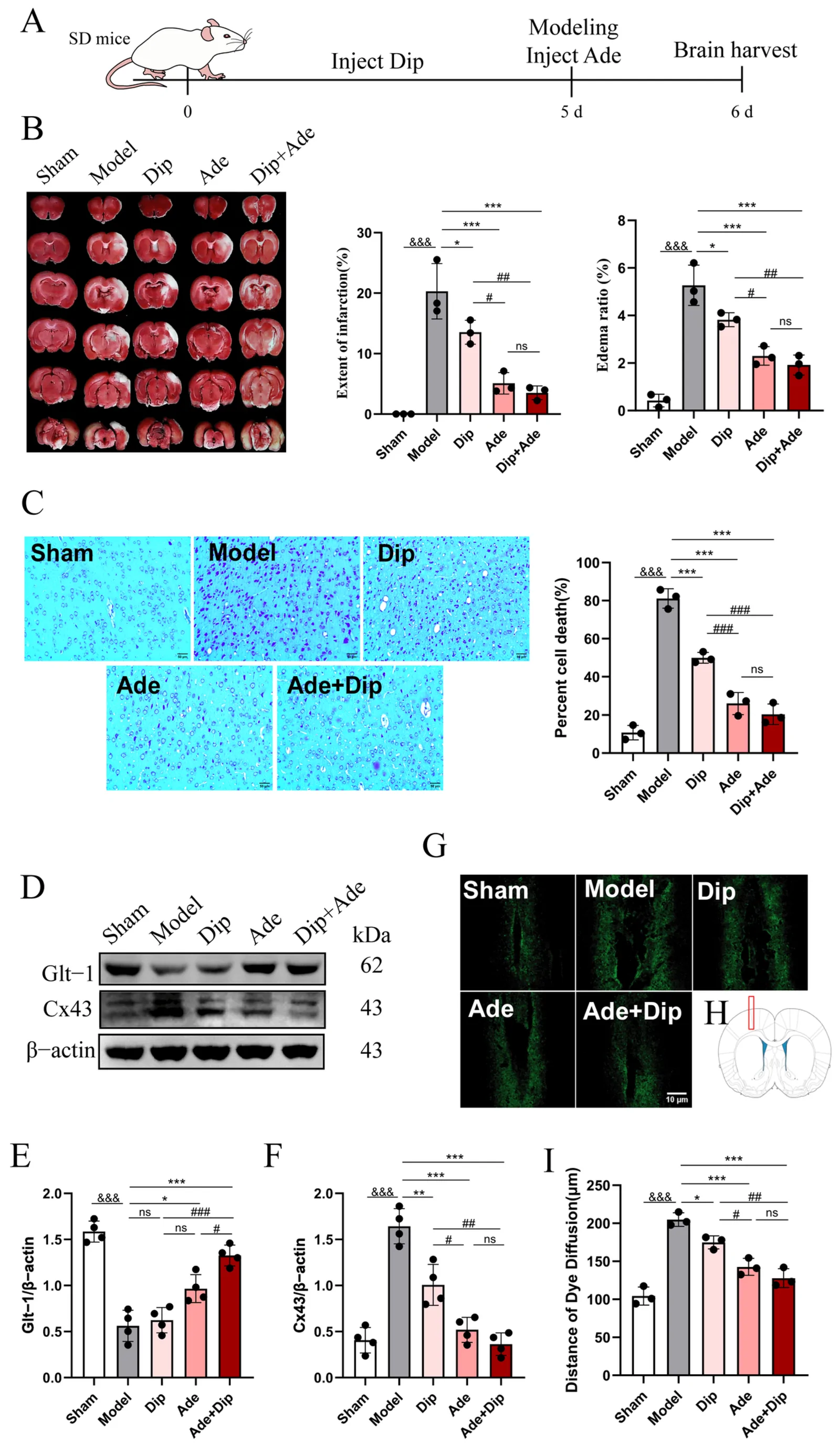
Exogenous adenosine demonstrates superior efficacy over endogenous adenosine in attenuating excitotoxicity. (**A**) Schematic illustration of the experimental design and procedural workflow. (**B**) TTC-stained brain tissue sections from the Sham, Model, Dip, Ade, and Dip+Ade groups of rats, along with quantitative analysis of the extent of infarction and edema rate; *n* = 3 independent samples. (**C**) Nissl staining to assess neuronal damage in the right cerebral cortex of five groups of rats, with quantitative analysis of neuronal cell death rate in each group. Scale bar: 50 µm; *n* = 3 independent samples. (**D**) Western blot analysis of GLT-1, Cx43, and β-actin expression in the right cerebral cortex of rats from the Sham, Model, Dip, Ade, and Dip+Ade groups. *n* = 4 independent experiments. (**E**,**F**) Quantitative analysis of protein expression levels shown in (**D**). (**G**,**I**) Fluorescein dye transfer experiments were performed on the right cerebral cortex of five groups of rats; fluorescence intensity represents the diffusion distance of the dye in the cortex. Scale bar: 10 µm. (**H**) Injection site. (**I**) Dye diffusion distance for each group. Data are expressed as the mean ± SD. Statistical significance: vs. Sham, ^&&&^ *p* < 0.001; vs. Model, * *p* < 0.05, ** *p <* 0.01, *** *p* < 0.001; vs. Dip, ^#^ *p <* 0.05, ^##^ *p* < 0.01, ^###^ *p* < 0.001; ns, no statistically significant difference. One-way ANOVA was performed with Tukey’s post hoc test.

**Figure 3:**
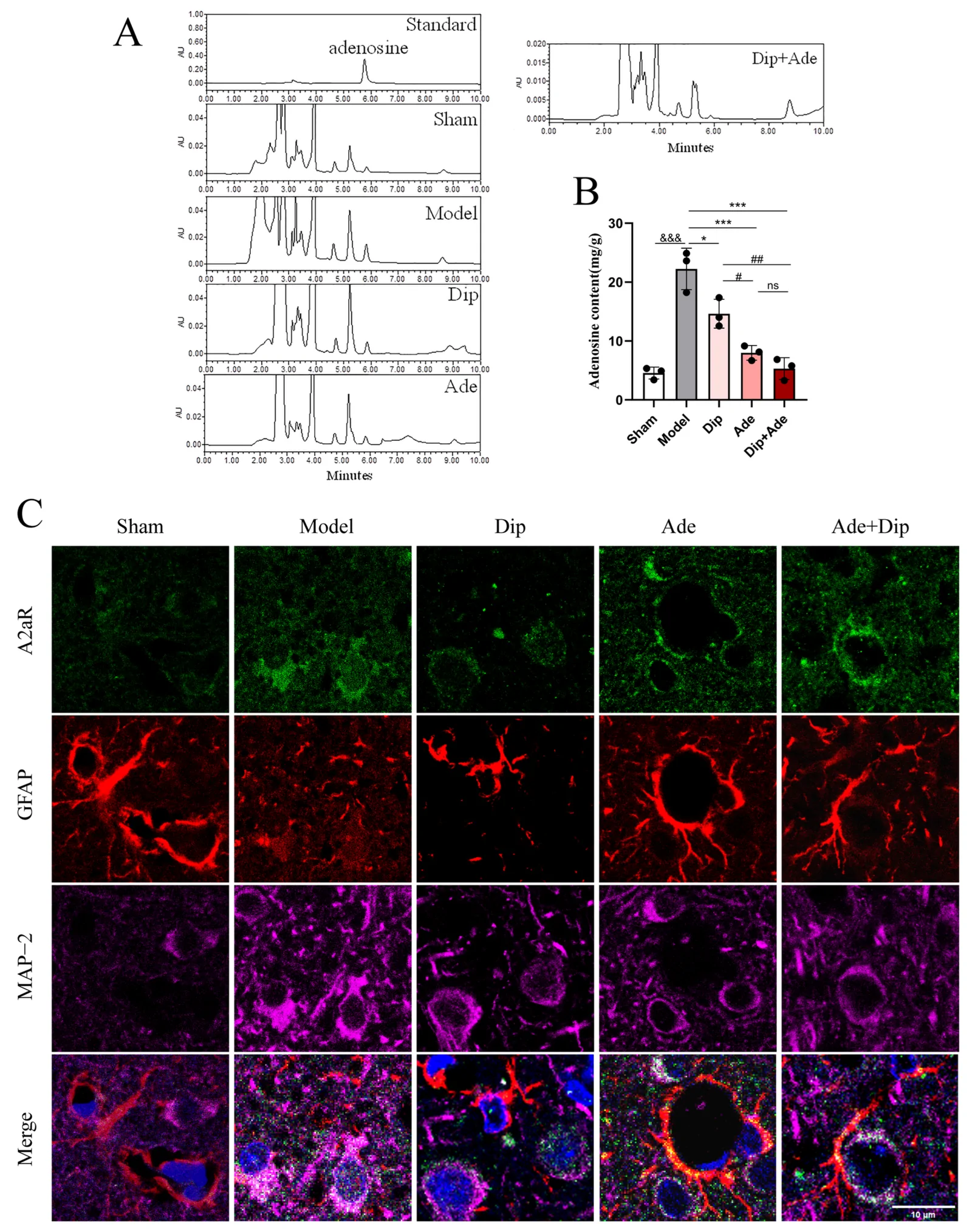
Adenosine may exert its effects by targeting the adenosine A_2A_R. (**A**,**B**) HPLC was used to determine the adenosine content in the rat cortex. The reference standard is adenosine, and its peak area is quantitatively proportional to concentration within the validated linear range. (**B**) Statistical analyses were performed using GraphPad Prism 5 software. (**C**) Immunofluorescence-based colocalization analysis was conducted on right cerebral cortical tissue sections from five experimental rat groups. DAPI (blue), A_2A_R (green), GFAP (red), and MAP-2 (magenta); scale bar = 10 µm; *n* = 3. Data are expressed as the mean ± SD. Statistical significance: vs. Sham, ^&&&^ *p* < 0.001; vs. Model, * *p* < 0.05, *** *p* < 0.001; vs. Dip, ^#^ *p* < 0.05, ^##^ *p* < 0.01; ns, no statistically significant difference. One-way ANOVA was performed with Tukey’s post hoc test.

**Figure 4:**
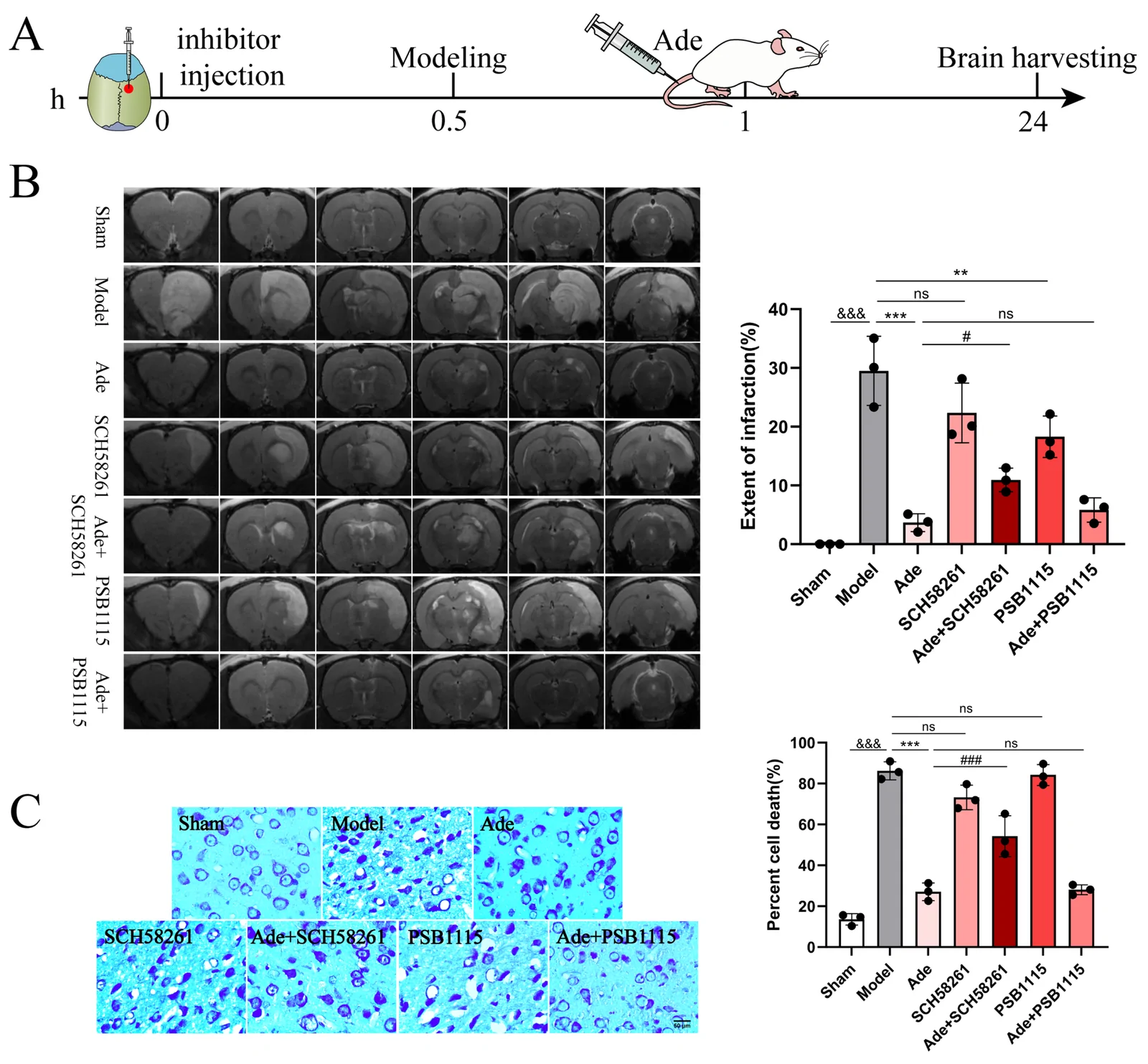
Inhibition of A_2A_R reverses the neuroprotective effects of adenosine. (**A**) Schematic illustration of the experimental design and procedural workflow. (**B**) Magnetic resonance imaging results and quantitative analysis of infarction extent in the brains of rats from seven groups: Sham, Model, Ade, SCH58261, Ade+SCH58261, PSB1115, and Ade+PSB1115. (**C**) Nissl staining to observe neuronal damage in the right cerebral cortex of seven groups of rats, with quantitative analysis of neuronal death rate in each group. Scale bar: 50 µm; *n* = 3. Data are expressed as the mean ± SD. Statistical significance: vs. Sham, ^&&&^ *p* < 0.001; vs. Model, ** *p* < 0.01, *** *p* < 0.001; vs. Ade, ^#^ *p* < 0.05, *^###^ p <* 0.001; ns, no statistically significant difference. One-way ANOVA was performed with Tukey’s post hoc test. To further identify the specific group comparisons underlying the observed differences, post hoc pairwise comparisons using the LSD test were conducted between Ade and Ade+SCH58261, and between Ade and Ade+PSB1115, based on the quantitative MRI data shown in panels (**B**,**C**).

**Figure 5:**
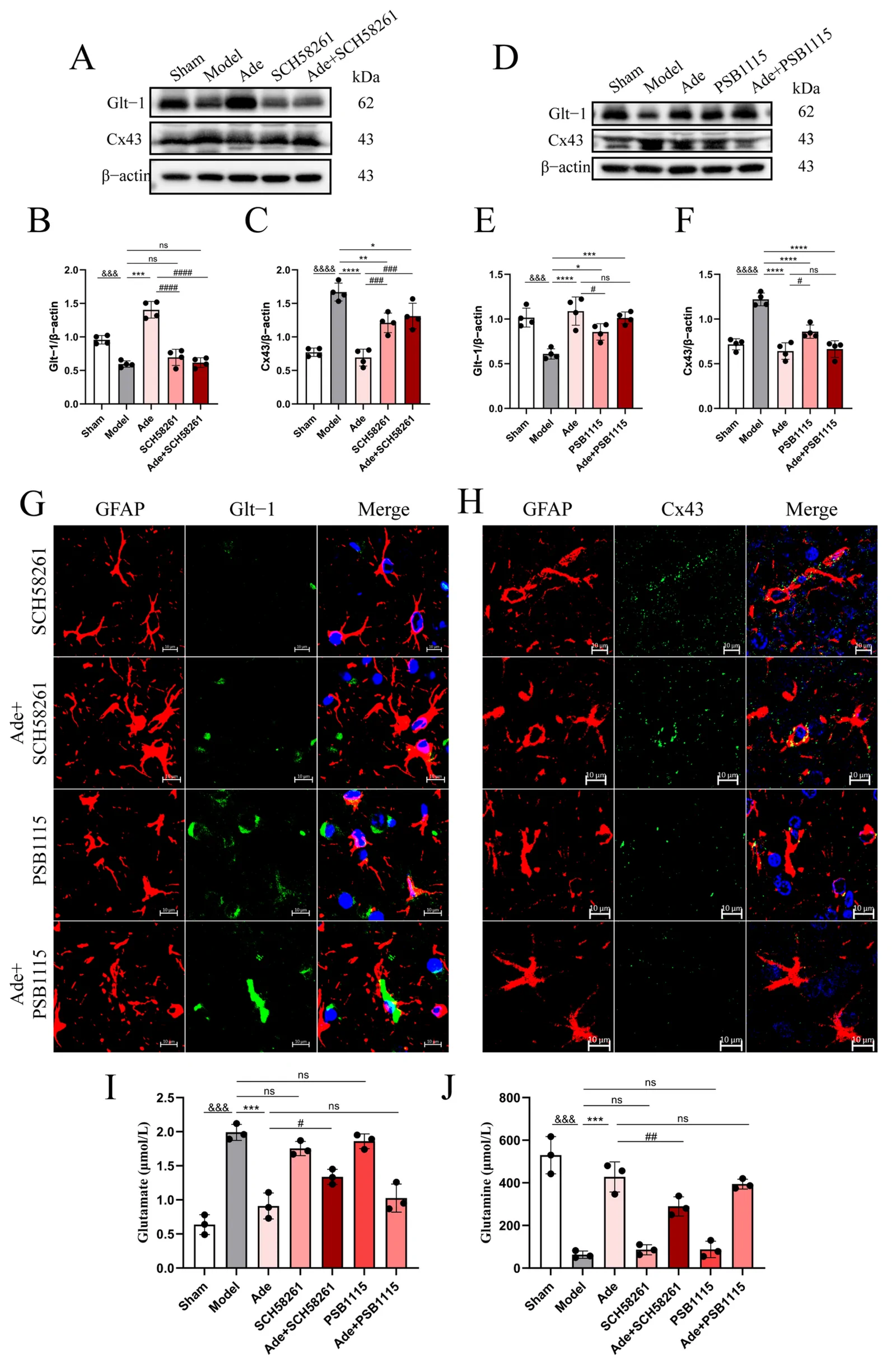
A_2A_R inhibition partially attenuates the excitotoxicity induced by adenosine receptor antagonism. (**A**) Western blot analysis of GLT-1, Cx43, and β-actin protein expression in the right cerebral cortex of rats from five groups: Sham, Model, Ade, SCH58261, and Ade+SCH58261; *n* = 4 independent experiments. (**B**,**C**) Quantitative analysis of protein expression levels shown in (**A**). (**D**) Western blot analysis of GLT-1, Cx43, and β-actin protein expression in the right cerebral cortex of rats from five groups: Sham, Model, Ade, PSB1115, and Ade+PSB1115; *n* = 4 independent experiments. (**E**,**F**) Quantitative analysis of protein expression levels shown in (**D**). (**G**,**H**) Immunofluorescence colocalization analysis of the right cerebral cortex in five groups of rats. DAPI: blue; GFAP: red; GLT-1 (**G**)/Cx43 (**H**): green. Scale bar: 10 µm. (**I**,**J**) Measurement of glutamate (**I**) and glutamine (**J**) levels in the right brain tissue of rats; *n* = 3. Data are expressed as the mean ± SD. Statistical significance: vs. Sham, ^&&&^ *p* < 0.001, ^&&&&^ *p* < 0.0001; vs. Model, * *p* < 0.05, ** *p* < 0.01, *** *p* < 0.001, **** *p* < 0.0001; vs. Ade, ^#^ *p* < 0.05, ^##^ *p* < 0.01, ^###^ *p* < 0.001, ^####^ *p* < 0.001; ns, no statistically significant difference. One-way ANOVA was performed with Tukey’s post hoc test. To further identify the specific group comparisons underlying the observed differences, post hoc multiple comparisons were conducted using the LSD test for glutamine levels (**J**): Ade vs. Ade+SCH58261, and Ade vs. Ade+PSB1115.

**Figure 6:**
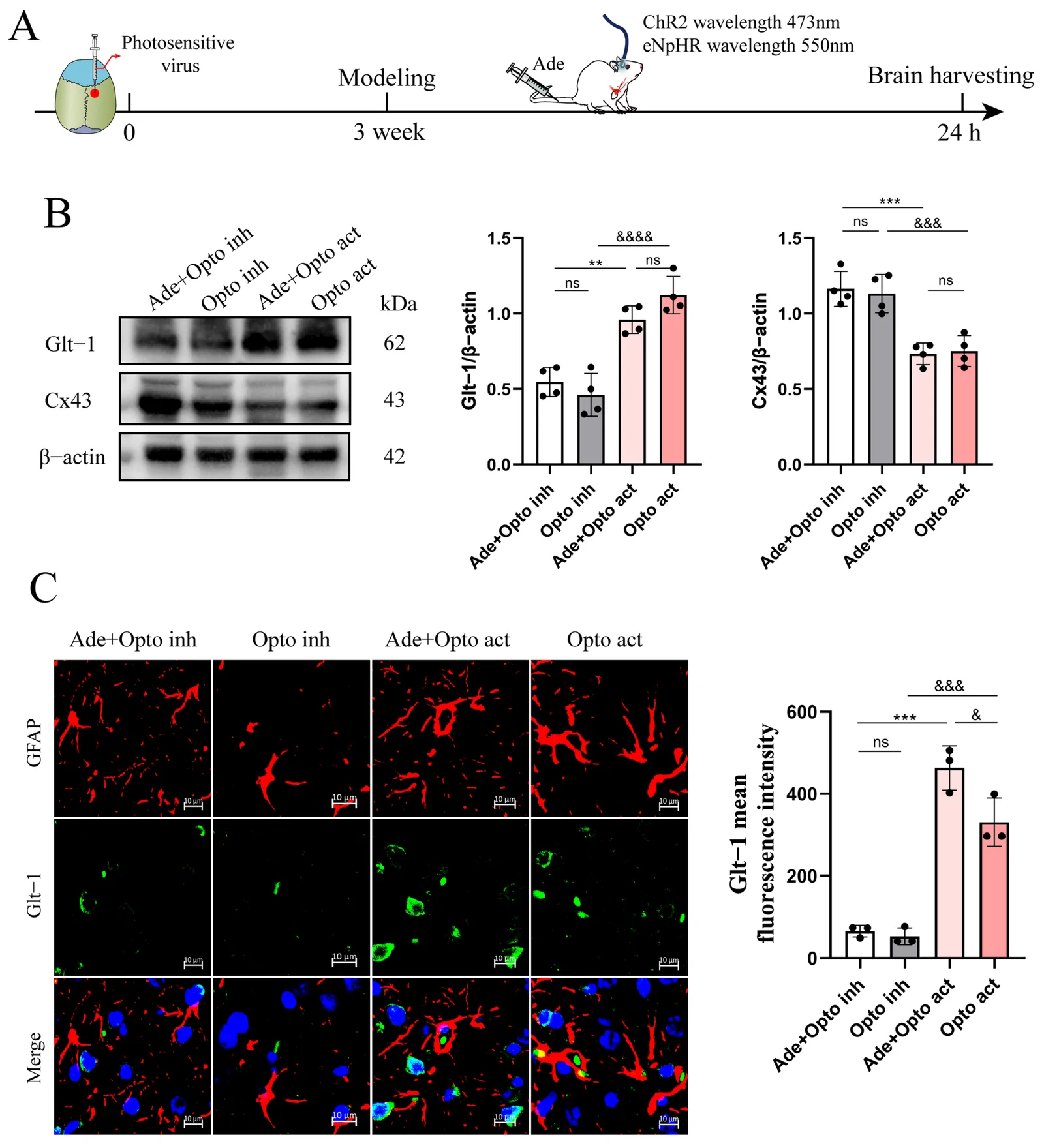
Astrocytes serve as key cellular targets mediating adenosine-dependent regulation of glutamate homeostasis. (**A**) Schematic illustration of the experimental design and procedural workflow. (**B**) Western blot analysis and quantitative assessment of GLT-1, Cx43, and β-actin protein expression in the right cerebral cortex of rats from four groups: Ade+Opto inh, Opto inh, Ade+Opto act, and Opto act; *n* = 4 independent experiments. (**C**) Immunofluorescence colocalization and quantification of GLT-1 and GFAP expression in the right cerebral cortex across four experimental groups. DAPI: blue; GFAP: red; GLT-1: green; scale bar: 10 µm; *n* = 3 independent biological replicates. Data are expressed as the mean ± SD. Statistical significance: vs. Ade+Opto inh, ** *p* < 0.01, *** *p* < 0.001; vs. Opto act, ^&^ *p* < 0.05, ^&&&^ *p* < 0.001, ^&&&&^ *p* < 0.0001. ns, no statistically significant difference. One-way ANOVA was performed with Tukey’s post hoc test.

## Data Availability

The original contributions presented in this study are included in the article/Supplementary Material. Further inquiries can be directed to the corresponding author.

## Supplementary Materials

The following supporting information can be downloaded at: https://www.mdpi.com/article/10.3390/cells15201915/s1. Figure S1: Adenosine significantly attenuates ischemia-induced excitotoxicity in the rat brain. Figure S2: Exogenous adenosine demonstrates superior efficacy over endogenous adenosine in attenuating excitotoxicity. Figure S3: Adenosine may exert its effects by targeting the adenosine A2AR. Figure S4: Inhibition of A2AR reverses the neuroprotective effects of adenosine. Figure S5: A2AR inhibition partially attenuates the excitotoxicity induced by adenosine receptor antagonism. Figure S6: Astrocytes serve as key cellular targets mediating adenosine-dependent regulation of glutamate homeostasis.
